# Impact of informed-choice invitations on diabetes screening knowledge, attitude and intentions: an analogue study

**DOI:** 10.1186/1471-2458-10-768

**Published:** 2010-12-17

**Authors:** Eleanor Mann, Ian Kellar, Stephen Sutton, Ann Louise Kinmonth, Matthew Hankins, Simon Griffin, Theresa M Marteau

**Affiliations:** 1 Psychology Department (at Guy's), Health Psychology Section, 5th Floor Bermondsey Wing, Guy's Campus, London SE1 9RT, UK; 2 Department of Public Health and Primary Care, University of Cambridge, Forvie Site, Robinson Way, Cambridge, CB2 0SR, UK; 3 Division of Primary Care and Public Health and Institute of Postgraduate Medicine, Brighton and Sussex Medical School, Falmer, BN1 9PH, UK; 4 MRC Epidemiology Unit, Institute of Metabolic Science, Box 285, Addenbrooke's Hospital, Hills Road, Cambridge, CB2 0QQ, UK

## Abstract

**Background:**

Despite concerns that facilitating informed choice would decrease diabetes screening uptake, 'informed choice' invitations that increased knowledge did not affect attendance (the DICISION trial). We explored possible reasons using data from an experimental analogue study undertaken to develop the invitations. We tested a model of the impact on knowledge, attitude and intentions of a diabetes screening invitation designed to facilitate informed choices.

**Methods:**

417 men and women aged 40-69 recruited from town centres in the UK were randomised to receive either an invitation for diabetes screening designed to facilitate informed choice or a standard type of invitation. Knowledge of the invitation, attitude towards diabetes screening, and intention to attend for diabetes screening were assessed two weeks later.

**Results:**

Attitude was a strong predictor of screening intentions (β = .64, p = .001). Knowledge added to the model but was a weak predictor of intentions (β = .13, p = .005). However, invitation type did not predict attitudes towards screening but did predict knowledge (β = -.45, p = .001), which mediated a small effect of invitation type on intention (indirect β = -.06, p = .017).

**Conclusions:**

These findings may explain why information about the benefits and harms of screening did not reduce diabetes screening attendance in the DICISION trial.

## Background

Invitations for screening have traditionally focused on maximising uptake, providing only information about population benefits [1]. Recent health policy in the UK and elsewhere has shifted towards the view that participation in screening should reflect individual informed choices, informed about the potential harms as well as individual benefits of screening [2,3]. In 2009, patients' right to information and choice about their healthcare became a legal right [4]. Informed choices can be considered to have two core characteristics: first, they should be informed by best current evidence; and second, they should reflect the decision-maker's values [5,6]. In the case of diabetes, the likelihood of health benefits for individuals arising from screening is low, and there have been concerns that telling people about the limited benefits and possible harms will reduce screening uptake [1,7]. At a population level this may increase the burden on the healthcare system as people with undiagnosed and therefore untreated diabetes have increased risk of developing complications. These concerns that patient informed choice might reduce screening uptake may explain why the informed choice policy is not generally implemented in screening. However, the veracity of this concern remains unknown [7].

The public health benefits of earlier detection and treatment of type 2 diabetes as a result of screening are uncertain [8,9]. The most efficient method of screening is likely to incorporate a step-wise process targeting those at highest risk as opposed to inviting everyone directly for a diagnostic test [10]. A step-wise procedure screens out many of those unlikely to have diabetes, but results in a high false positive rate at initial testing; most of the people invited back for further testing will not have diabetes at that time.

According to the Theory of Planned Behaviour [11], which has been widely applied to screening participation, salient beliefs about the personal consequences of the target behaviour determine attitude toward the behaviour which in turn influences intention to perform that behaviour; to the extent that the behaviour is under volitional control, intention is a proximal determinant of behaviour. A recent meta-analysis [12] of studies using the theory of planned behaviour to predict screening uptake found that attitudes towards screening were a good predictor of screening intentions (r = .51, p < .001), and that intentions were a good predictor of screening uptake (r = .42, p < .001). If an increase in knowledge leads to changes in salient behavioural beliefs i.e. those that influence attitudes, then this may lead to changes in intentions and behaviour. Therefore if giving information about screening limitations leads to more negative salient beliefs about screening we would predict that fewer people would attend for screening.

DICISION is a randomised clinical trial of an informed choice invitation to diabetes screening. It aimed to test the validity of concerns that facilitating informed choices would lead to reduced attendance for screening, particularly in the more socially deprived. As part of the DICISION trial, an experimental analogue study was conducted in order to develop the informed choice invitation [13]. The invitation contained evidence-based information, in accordance with General Medical Council (GMC) guidelines [2], and was designed to be read and understood across a wide range of literacy. Rates of informed choice were significantly higher after reading the invitation designed to facilitate informed choice compared to a standard one, similar to those currently used. The invitation was then used in a clinical trial of diabetes screening uptake [14]. In the main trial no difference in screening uptake was found between participants who received the informed choice invitation and those receiving the standard invitation [15]. Impacts of informed choice on screening uptake might be expected to vary depending on the condition screened for. For example, some screening tests can entail physical harms that include disability, e.g. prostate cancer [16], whereas the potential harms of diabetes screening may be considered less harmful, for example, unnecessary worry as a result of false positive test results. Mathieu, Barratt, Davey et al [17] found no difference in breast cancer screening uptake in women receiving an informed choice decision aid compared to usual care. Trevena, Irwig and Barratt [18] found no impacts of a decision aid on rates of self reported use of colorectal cancer screening kits. By contrast Krist, Woolf, Johnson and Kearns [19] found fewer requests for prostate cancer screening tests following a decision aid. In the DICISION trial, to avoid possible measurement effects, questionnaire data were not collected until after the primary outcome (attendance) had been measured. However, data from the experimental analogue study can be used to model the impact of an invitation designed to foster choice on the cognitive antecedents of screening intentions and thus to explore possible explanations for the findings of the DICISION trial.

### The present study

The present study reports data from the experimental analogue study conducted prior to the DICISION trial. Intentions to attend for screening are used as the primary outcome. Although screening intentions do not equate to actual attendance, they are a good predictor [12]. The analogue design also ensures that all participants view the invitation, which could not be controlled for in the clinical trial, so this study tests whether the materials used in the DICISION trial manipulate cognitions as hypothesised and serves as an explanatory account of the objective outcomes of the DICISION trial. The impact on rates of informed choice of the invitation developed for the trial, and cognitive differences by invitation type were reported by Kellar et al [13]. The present study tests whether invitation type impacts on intention, mediated by attitude, as would be predicted by the theory of planned behaviour.

### Objective

To test a model of the impact on knowledge, attitude and intentions of a diabetes screening invitation designed to facilitate informed choices.

## Methods

### Participants

Members of the public were approached by market research representatives in the street, in town centres around the UK between February and April 2006. Eligible participants were aged between 40 and 69 years, with no previous diagnosis of diabetes, who agreed to provide demographic details and accept a follow-up visit at their homes 2 weeks later. Additionally, a quota was set of 50% of participants having finished full-time education at 16 or before. All questionnaire measures were delivered verbally. 196 males and 221 females took part. They were told that they were at a higher risk of developing type 2 diabetes because they were 40 years old or over. Participants then viewed one invitation taken from the top of a randomly ordered pile (either standard or one of two versions of an informed choice invitation). The materials were ordered in a way that the invitation type was hidden until the recruitment process was completed. Moreover, interviewers were not aware of the direction of anticipated effect of materials, and materials were dummy-coded, so that no sense of intervention or control would have been communicated to interviewers or participants. After the participant had read the invitation they were told:

"[it is] *an invitation to attend a diabetes screening appointment. The appointment will not take place, but please vividly imagine that you have received this from your GP regarding a real appointment*".

Two weeks later the participants completed a verbally-administered questionnaire without referring back to the invitation, conducted at participants' homes by a market researcher. Participants received £5 on completion of the study.

The research was conducted in compliance with the Helsinki Declaration [20] and ethical approval was obtained from Cambridge University Ethics Committee.

### Intervention materials

Two invitations to attend for diabetes screening were developed for this study: a standard invitation (control group), and an invitation designed to facilitate informed choice (see additional files 1 and 2). Two versions of the informed choice invitation were developed. In the first, participants were asked to list "good things" and "bad things" about screening for diabetes. In the second, participants were asked to list "good feelings" and "bad feelings". There were no significant effects of this manipulation and the two groups were treated as a single group in the analysis reported here.

### Standard invitation

The standard invitation, shown in additional file 1, was based upon invitations commonly used to invite people for diabetes and coronary heart disease screening [21]. It presented a brief didactic argument, describing only benefits of attending for screening. It explained that the participant might have a higher chance of developing type 2 diabetes, and that diabetes has serious long term consequences.

### Informed choice invitation

The informed choice invitation, shown in additional file 2, contained the information described above, plus information which included the limited benefits and potential harms of attending for screening. The text of the invitation explained both absolute risks and relative risk using frequencies, e.g. "If 100 people had the test, about 63 would get this result". Previous studies have shown that risk information is most readily understood using frequencies in this way [22]. Participants were encouraged to make a choice that reflected their values by prompting them to evaluate the consequences and asking them to record their decision to attend or not.

#### Providing information about diabetes risk and consequences of screening

This section was developed from the UK General Medical Council (GMC) guidelines for providing sufficient information when gaining patient consent [2]. These guidelines include purpose of screening, details of diagnosis and prognosis with and without treatment, probability of benefits and risks, and emphasis on patient choice. The invitation began with an emphasis on patient choice "*Screening for diabetes. It's your decision*", and a statement that the participant was being offered screening for type 2 diabetes because they might have a higher chance of developing the condition. An explanation of diabetes and the screening procedure followed, then an explanation of the expected results and what they mean for the patient. Finally, the benefits and harms of attending for screening were outlined, including likely prognosis of early treatment compared to standard treatment following clinical diagnosis and the potential for unnecessary worry following false positive results.

#### Encouraging participants to make a choice

At the end of the hypothetical invitation letter, participants were asked to consider the consequences of their attending diabetes screening and to indicate their decision as to whether to go for screening or not, or to think more about the decision.

The content and format of the informed choice invitation were refined through extensive piloting using "think aloud" techniques. Both invitations were designed to be comprehensible to those with a reading age of 11 or above (Flesch Reading Ease score was 71.52 and 72.88 for the standard and the informed choice invitations, respectively). Rates of informed choice were significantly higher after reading the informed choice invitation compared to the standard invitation [13].

### Measures

Gender, age group (40-49, 50-59, 60-69 years), and job title of the highest earner in the household were recorded. Job title was used to assign a social grade [23]: Grades A and B encompassed mid or top level management and other high level professionals, grade C1 included junior management and other non manual occupations, grade C2 included skilled manual workers, grade D included semi and unskilled workers, and grade E referred to the state dependent and those without regular employment.

Strength of intention to attend for screening (herein termed 'intention') was measured as a behavioural expectation using two items: "Would you have the diabetes screening test?" (5 point scale; 1 - definitely no, 2 - probably no, 3 - not sure, 4 - probably yes; 5 - definitely yes), and "How likely is it that you would have the diabetes screening test?" (7 point scale from 1 - extremely unlikely to 7 - extremely likely). An strength of intention scale with a zero mid point was constructed by subtracting four from responses to the 7-point scale (-3 to 3) and three from the 5-point scale (-2 to 2). The 5-point scale was converted to a scale equivalent to the 7-point scale by multiplying the values by 7/5 (-2.8 to 2.8). Subsequently individuals' scores for the two scales (r = .76, p < .001) were summed resulting in a scale ranging from -5.8 to 5.8.

Attitude was indexed by the mean of six items adapted from Marteau et al [6] measured on 7-point scales: "For me, having the screening test for diabetes would be..." (not worthwhile - worthwhile; unimportant - important; harmful - not harmful; not beneficial-beneficial; not a good thing - a good thing; a bad thing - not a bad thing) (Cronbach's α = .88). Higher attitude scores indicate more positive attitude (range: 1 to 7)

Eight multiple choice items measured participants' knowledge of the implications of diabetes screening. These items were developed for this study based on GMC guidelines for providing information about screening [2]. Examples included "What are the possible harms of screening for diabetes?", "How effective is early treatment for diabetes in preventing long term problems?", and "For most people, what is the most likely test result from diabetes screening?" (see additional file 3). Higher knowledge scores indicate better knowledge (Range 1 to 8).

### Analysis

T tests are used to describe the impacts of the informed choice invitation on knowledge, attitudes and intentions. Pearson's correlations are used to explore the associations between knowledge, attitudes and intentions. 400 participants are needed for 80% power to detect a small effect (d = .3) of the informed choice invitation on knowledge, attitudes and intentions (assuming an alpha of .05 and a two-sided test).

The impact of the invitations on strength of intentions was modelled using AMOS 7. A path model was specified in which invitation type influenced knowledge, which influenced attitude, which in turn influenced intention. No other paths were specified. A maximum likelihood bootstrapping procedure (2000 samples) was used to estimate the model. Model fit was tested with the chi square test (χ^2^), root mean square error of approximation (RMSEA), the comparative fit index (CFI), and the standardized root mean square residual (SMSR). Good fit is indicated when χ^2 ^is non-significant (although this is rare in samples over 100), RMSEA is less than .05 (although close fit for RMSEA is <.08 [24]), CFI is greater than .95 and SMSR is less than .08 [25]. In addition, a multiple group analysis was conducted to test whether the model relating knowledge, attitude and strength of intention differed in the two invitation groups. This was done by constraining the regression coefficients to be the same in the two groups and examining the reduction in fit compared with a model in which these parameters were freely estimated in each of the two groups.

## Results

Participants' demographic characteristics are summarised in Table 1. The modal age group was 40-49 and 53% (221) were female. The modal social grade was C1 (supervisory or clerical, junior managerial, administrative or professional), reflecting national data, but overall this sample had a higher proportion of less deprived participants than that found in the UK population [26].

**Table 1:** Demographic characteristics of the study groups (n = 417).

	Invitation Type	
	**Informed choice; n = 278 N (%)**	**Standard; n = 139 n (%)**

Age range		

40-49	140 (50.4)	66 (47.5)
50-59	90 (32.4)	40 (28.8)
60-69	48 (17.3)	33 (23.7)

Gender		

Female	152 (54.7)	69 (49.6)
Male	126 (45.3)	70 (50.4)

Social Grade		

A or B	91 (32.7)	35 (25.2)
C1	104 (37.4)	52 (37.4)
C2	59 (21.2)	29 (20.9)
D	19 (6.8)	20 (14.4)
E	5 (1.8)	3 (2.2)

139 participants viewed the standard invitation to attend for diabetes screening and 278 participants viewed one of the invitations designed to facilitate informed choice. Five participants from the standard group and five participants from the informed choice group did not complete follow up measures two weeks later, resulting in 134 in the standard group and 273 in the informed choice group (n = 407, 98% response rate). There were no differences in age between the standard and the informed choice group (χ^2 ^(1) = 2.47, p = .29), sex (χ^2 ^(1) = .76, p = .39) or social grade (χ^2 ^(1) = 2.65, p = .11).

Means and standard deviations for knowledge, attitudes and intentions in the two invitation groups are shown in Table 2. Those who received the informed choice invitation showed greater knowledge of the potential benefits and harms of screening than those who received the standard invitation (difference in means = 1.61 (95%CI: 1.29 to 1.92), p < .001), whereas attitude did not differ significantly (difference in means = .11 (95%CI: -.08 to .30), p = .27). Intentions to attend for screening were slightly stronger in the informed choice group, but the difference did not reach significance (difference in means = .49 (95%CI: -.03 to 1.01), p = .07). Rates of informed choice and group differences in knowledge and attitudes were originally reported in Kellar et al [13].

**Table 2:** Knowledge, attitudes and intentions (mean (SDs)) overall and in each invitation group.

	Overall (n = 407)	Informed choice (n = 273)	Standard (n = 134)
Knowledge	4.97 (1.69)	5.49 (1.53)	3.89 (1.47)
Attitude	6.21 (.92)	6.25 (.89)	6.14 (.98)
Intention	4.01 (2.38)	4.17 (2.22)	3.68 (2.65)

Higher knowledge scores indicate better knowledge of screening (range: 1 to 8); Higher attitude scores indicate more positive attitudes towards attending for screening (range 1 to 7); higher intention scores indicate stronger intentions to attend for screening (-5.8 to + 5.8)

Correlations between knowledge, attitudes and intentions are shown in Table 3. Knowledge and attitude were uncorrelated (r = .01, p = .88), whereas there was a small positive correlation between knowledge and intention (r = .13, p = .008). As expected, there was a large correlation between attitude and intention (r = .64, p < .001). Correlations did not vary much by invitation type; there was a significant correlation between knowledge and intention in the informed choice group (r = .15, p = .01) but not in the standard group (r = .01, p = .94). However, the difference in correlation size was not significant (z of difference = 1.33, p = .18).

**Table 3:** Correlations (Pearson's r) between knowledge, attitudes and intentions overall and by group.

	Overall (n = 407)	Informed choice (n = 273)	Standard (n = 134)
Knowledge-Attitude	.01	.00	-.06
Knowledge-Intention	.13**	.15*	.01
Attitude-Intention	.64***	.61***	.70***

* p < .05; ** p < .01; *** p < .001

**Figure 1:**
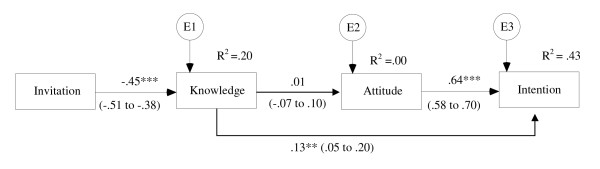
**Model of the impact of the informed choice invitation on screening intentions 2 weeks later (n = 407)**. Notes: *** p = .001, ** p = .005. Model fit: χ^2^(2) = 1.35, p = .51; CFI = 1.00; RMSEA = .00, SMSR = .02. 90% confidence intervals for standardised regression weights shown in brackets Negative β weights between invitation and knowledge indicate that the informed choice invitation was associated with higher knowledge. Indirect effect of invitation on intention = -.06 (90%CI: -.10 to -.02), p = .017. Indirect effects of invitation on attitude = .003 (90%CI: -.04 to .03), p = .853

A model of screening intentions was specified in which invitation type predicted knowledge, which predicted attitudes, which predicted strength of intentions. Fit could be considered adequate: although chi square was significant (χ^2^(3) = 12.68, p = .005) this is likely to reflect the large sample size rather than poor fit; RMSEA was greater than.08 (RMSEA = .09), but CFI was greater than .95 and SMSR was less than.08 (CFI = .97; SMSR = .05). The path from knowledge to attitude was non-significant (β = .01, p = .88), and modification indices suggested a direct path from knowledge to intention. This path was added, and the resulting model (shown in figure 1) was an excellent fit to the data (χ^2^(2) = 1.35, p = .51; CFI = 1.00; RMSEA = .00; SMSR = .02). Modification indices did not indicate that the model could be improved by adding further paths (i.e. a direct path from invitation to attitude or intention). Invitation type was a good predictor of knowledge (β = -.45, p = .001), but did not impact upon attitude either directly (as indicated by model fit and modification indices) or indirectly mediated by knowledge (indirect effects: β = .003 (90%CI -.04 to .03), p = .853). 43% of the variance in intention was mainly explained by attitudes (β = .64, (90%CI: .58 to .70), p = .001), but knowledge also had a small direct impact on intention (β = .13 (90%CI: .05 to .20), p = .005), which was unmediated by attitudes. The indirect effect of the invitation on intention, mediated by knowledge, was small but significant (indirect β = -.06 (90%CI: -.10 to -.02), p = .017). The direct effect of knowledge on intention appeared to be driven by the informed choice invitation, as there was no correlation between knowledge and intention in the standard group (r = .01, p = .94). A multiple group analysis was then conducted, comparing the model (figure 1) separately for the two groups. These showed no significant reduction in fit when the regression coefficients for attitude and knowledge were constrained to be the same in the two invitation groups (χ^2^(2) = 4.83, p = .09), suggesting that the same model was applicable to the standard group as to the informed choice group.

## Discussion

These results suggest that informed choice invitations that increase knowledge alone have little effect on attitudes or strength of intentions to attend for screening. Attitudes and intentions to attend for screening were strong regardless of invitation type. Invitation type had a small indirect effect on intention, mediated by knowledge, but attitudes towards screening were the main predictor of intention, and were unaffected by invitation type.

These results suggest that the informed choice invitation did not reduce strength of screening intentions because it did not result in more negative attitudes towards screening. Attitudes were the main predictor of screening intentions, mirroring findings in a recent meta-analysis [12]. However, from the standpoint of the Theory of Planned Behaviour, an increase in knowledge would not necessarily be expected to produce a change in salient beliefs, partly because the recipient might not apply the information to their own specific case e.g. "knowing" that many people do not benefit from screening does not mean that I think I will not benefit from screening. Even if an increase in knowledge does produce changes in salient beliefs, these changes may not all be in the same direction i.e. an increase in knowledge could produce an increase or a decrease in attitude. Our findings are generally consistent with those of previous studies. A review by Fox [27] found few studies of informed choice materials in which changing knowledge resulted in changes in attitudes. Of nine trials of written information, eight assessed knowledge and four assessed attitudes. While five out of eight trials assessing knowledge showed increased knowledge, only one of those assessing attitudes showed any change in attitude (towards more negative screening attitudes [28]). Concerns that facilitating informed choice will reduce screening uptake seem to be based upon the unfounded assumption that providing information changes behaviour. At best, information-based interventions have shown mixed effects on behaviour [27,29,30]. We did find a small indirect effect of the informed choice invitation on intention, mediated by a positive effect of knowledge. It was not, however, large enough to translate into significant invitation group differences. Either the effect was a chance finding or it was a robust effect too small to be of applied value.

Using the same invitations, the DICISION trial found no differences in attendance for screening. Thus the present study adds to the growing body of evidence that providing information designed to facilitate informed choice is unlikely to have significant detrimental impacts upon behaviour. However, whilst the invitation may have increased knowledge sufficiently, it may have failed to facilitate choice. The present study reported positive attitudes and strong intentions to attend for screening. By contrast, in the DICISION trial, uptake of screening was lower, particularly in those with high levels of social deprivation. This suggests that screening attendance may have been driven more by practical barriers than by cognitive differences. Such findings would indicate that participants did not accurately envisage the practical barriers they would face if actually invited for screening, rather than reflecting low motivation to attend. Saidi, Sutton and Bickler [31] found practical barriers were the most commonly reported reason for non-attendance in the unemployed. Most interventions designed to facilitate informed choices concentrate on increasing knowledge only. Future interventions need to consider how best to enable people to act in accordance with their intentions.

The potential benefits and harms of screening vary widely depending on condition screened for, which may influence the impacts of informed choice. As a result the generalisability of the findings to screening for other conditions is unknown. The harms that can arise from screening for diabetes are generally not considered serious [32,33] and were described in the invitations we used as comprising worry prior to an appointment and false reassurance following a "screen negative" test result. In contrast, undergoing other screening tests can entail physical harms that include disability and even death e.g. colonoscopy [34]. Evidence of the impact of knowledge about such potential harms and the uncertain limited individual benefit of screening is mixed [27,30]. It is important to study screening for different conditions, in order to find out whether it is valid to talk about the impacts of informed choice on screening uptake in general, or whether we should treat screening tests for different conditions separately.

If making informed choices does not change decisions at all, is there a benefit of investing resources in facilitating informed choice? Informed choice might not change decisions, but instead increase well-being or have other beneficial effects. Evidence suggests that better knowledge of screening reduces anxiety in those recalled for further testing [35]; although diabetes screening does not seem to cause high levels of anxiety [32]. More research into the cost-effectiveness of facilitating informed choice may be needed, but setting the threshold at which the costs of facilitating informed choice outweigh the benefits is a value judgement.

There are several limitations to this study. Although the present sample was at risk of diabetes because they were over 40, they were not necessarily representative of the highest risk individuals in this age group. Only people who visited town centres during normal office hours and would accept a home visit for follow up were included, and £5 incentive may have also added a selection bias. The lack of anonymity with verbally delivered questionnaires might encourage socially desirable responding, but use of neutral market researchers may reduce this effect, compared to, for example, a member of the study research team. The drawbacks of an analogue design must be acknowledged. We make the assumption that intentions obtained through imagining a hypothetical screening scenario are a good indication of actual attendance for screening. Although intentions are the best predictor of screening uptake overall [12], intentions to attend for screening were higher than actual uptake found in the clinical context of the main DICISION trial. Furthermore, we suggest an explanatory account of the DICISION trial findings because we test the same intervention materials, but the two studies are independent, using separate samples of participants, and therefore our conclusions are tentative pending verification in a clinical population. Finally, our conclusions are generalisable only to diabetes screening until further research indicates to what extent impacts of informed choice in screening are dependent upon the context.

## Conclusions

An invitation designed to facilitate informed choice that increased knowledge alone did not affect intentions, because it did not affect attitudes, the main predictor of intentions. Attitudes and intentions to attend for screening were strong regardless of information received. These findings add to a growing body of evidence showing that providing information about the potential benefits and harms of attending for screening will not reduce screening uptake and therefore does not conflict with maximising population health. However, facilitating informed choice may require interventions that bridge the intention-action gap in screening uptake, particularly among socially deprived groups.

## Competing interests

The authors declare that they have no competing interests.

## Authors' contributions

EM conducted statistical analysis and drafted the manuscript; IK is the study coordinator; MH provided statistical analysis support; TMM, ALK, SG and SS are Principal Investigators; TMM is the paper guarantor. All authors read and approved the final manuscript.

## Pre-publication history

The pre-publication history for this paper can be accessed here:

http://www.biomedcentral.com/1471-2458/10/768/prepub

## Supplementary Material

Additional file 1 **The standard invitation**. The invitation received by participants in the control arm Click here for file

Additional file 2 **The informed choice invitation**. The invitation received by participants in the intervention arm Click here for file

Additional file 3 **Multiple-choice diabetes screening knowledge questionnaire**. The 8 items that comprise the multiple-choice diabetes screening knowledge questionnaire Click here for file
