# Supplementary material for: Impact of informed-choice invitations on diabetes screening knowledge, attitude and intentions: an analogue study
Source: BMC Public Health. 2010 Dec 17;10:768. doi: 10.1186/1471-2458-10-768 (PMC3019193; doi:10.1186/1471-2458-10-768)
Supplement: Additional file 3 — Multiple-choice diabetes screening knowledge questionnaire. The 8 items that comprise the multiple-choice diabetes screening knowledge questionnaire [file 1471-2458-10-768-S3.DOC]

Diabetes is?

(CIRCLE ONE CODE ONLY)

A serious condition

A mild condition

I don’t know

Common long term problems from diabetes include?

(CIRCLE ONE CODE ONLY)

Dry skin

Headaches

Heart disease

Cancer

Colds and flu

I don’t know

The screening test for diabetes is? (CIRCLE ONE CODE ONLY)

A finger prick test

A test where blood is taken from a vein in the arm

A urine test

A fitness test

I don’t know

For most people, what is the most likely test result from diabetes screening?

(CIRCLE ONE CODE ONLY)

Definitely do not have diabetes

Probably do not have diabetes

Probably do have diabetes

Definitely do have diabetes

None of these

I don’t know

If your screening result says “You do not have diabetes now”, what does this mean?

(CIRCLE ONE CODE ONLY)

Definitely do not have diabetes

Probably do not have diabetes

Probably do have diabetes

Definitely do have diabetes

None of these

I don’t know

If further tests show that a person definitely does have diabetes, what do you think they would be offered?

(CIRCLE ALL CODES THAT APPLY)

Medicine

Another type of test

Advice about diet and exercise

Extra vitamins

None of these

I don’t know

How effective is early treatment for diabetes in preventing long term problems? (CIRCLE ONE CODE ONLY)

Everyone benefits

Most people benefit

A few people benefit

No-one benefits

I don’t know

What are the possible harms of screening for diabetes?

(CIRCLE ONE CODE ONLY)

People told that they do not have diabetes now may think that they can lead a less healthy life.

The test can cause dry skin

The test can cause skin allergies

People told that they may have diabetes now are more likely to have headaches.

None of these

I don’t know
